# The surge of HBsAb level in a HBsAg-negative ES-SCLC patient after anlotinib plus atezolizumab treatment: A case report

**DOI:** 10.3389/fonc.2023.1103512

**Published:** 2023-04-18

**Authors:** Gangjun Chen, Tian Tian, Xingdong Cai

**Affiliations:** Department of Respiratory, The First Affiliated Hospital of Jinan University, Guangzhou, China

**Keywords:** anlotinib, atezolizumab, small-cell lung cancer, HBsAb level, HBV functional cure

## Abstract

Small-cell lung cancer (SCLC) is a poorly differentiated neuroendocrine tumor with endocrine function. For decades, chemotherapy and immune checkpoint inhibitors (ICIs) have been the first-line treatment options. Because of its ability to normalize tumor vessels, anlotinib is recommended as a novel therapy as a third-line treatment. A combination of anti-angiogenic drugs and ICIs can effectively and safely benefit advanced cancer patients. However, immune-related side effects caused by ICIs are common. Hepatitis B virus (HBV) reactivation and hepatitis are common during immunotherapy in patients with chronic HBV infection. A 62-year-old man with ES-SCLC who had brain metastasis was described in this case. It is unusual for a HBsAg-negative patient to develop an increase in HBsAb after receiving atezolizumab immunotherapy. Although some researchers have reported the functional cure of HBV by PD-L1 antibody, this is the first case that showed a sustained increased in HBsAb level after anti-PD-L1 therapy. It is related with CD4+ and CD8+ T cells activation and HBV infection microenvironment. Importantly, this could provide a solution to insufficient protective antibody production after vaccination as well as a therapeutic opportunity for HBV patients with cancers.

## 1 Introduction

Extensive-stage small cell lung cancer (ES-SCLC) is a highly malignant and aggressive lung cancer subtype with a poor prognosis. It accounts for approximately 15% of lung cancer cases (1, 2). Relapse is very common during treatment after traditional systemic chemotherapy and radiotherapy (3). Immune checkpoint inhibitors (ICIs) and anti-angiogenic agents are novel strategies for ES-SCLC (4). Programmed death-ligand 1(PD-L1) and programmed death 1(PD-1) are commonly observed in human cancers. Attention is focused on the research of the efficacy and safety of anti-angiogenic drugs combined with PD-1/PD-L1 inhibitors (5, 6).

Anti-angiogenic drugs can stimulate antigen presentation and activate cytotoxic CD+8 T cells, lymphocyte infiltration and migration, hence lowering immunosuppression (7). Moreover, by alleviating immunosuppression, PD-1/PD-L1 antibodies can also increase the anti-cancer effects of anti-angiogenic agents (4). Anlotinib is a small-molecule receptor tyrosine kinase (RTK) that inhibits tumor neovascularization and cell migration (8). Moreover, many clinical trials and case reports provided sufficient proofs for combination of anti-angiogenesis medications and ICIs in ES-SCLC patients (9, 10). A real-world retrospective study in China had found a significantly longer PFS in patients with relapsed SCLC who received anlotinib plus PD-1 inhibitor than those who received PD-1 inhibitor alone (n=14,5.0 vs.3.0 months; p=0.005) (11).

Unfortunately, even though ICIs increase the natural killing response against tumor cells, immune-related adverse events (irAEs) can lead to the suspension of ICIs and even death in patients (12). The spectrum of irAEs caused by anti-PD-1 and PD-L1 is extensive, including pneumonia (35%), hepatitis (22%), enteritis (17%), nervous system toxicity (15%), and myocarditis (8%) (13). Hepatitis caused by immunotherapy is usually occurred in chronic hepatitis B virus (HBV) infected patients. HBV reactivation also happens in HBsAg-positive patients undergoing anti-PD-1/PD-L1 therapy (14). But the continuous increase of protective HBsAb in HBsAg-negative cancer patients has never been found.

Here we reported a HBsAg-negative patient with ES-SCLC whose HBsAb increased persistently during the combination of anlotinib with atezolizumab treatment. This combination therapy has brought satisfactory survival outcome. We try to explain the reason for this odd phenomenon and hope to find functional cure strategy for HBV patients.

## 2 Case presentation

**Figure 1:**
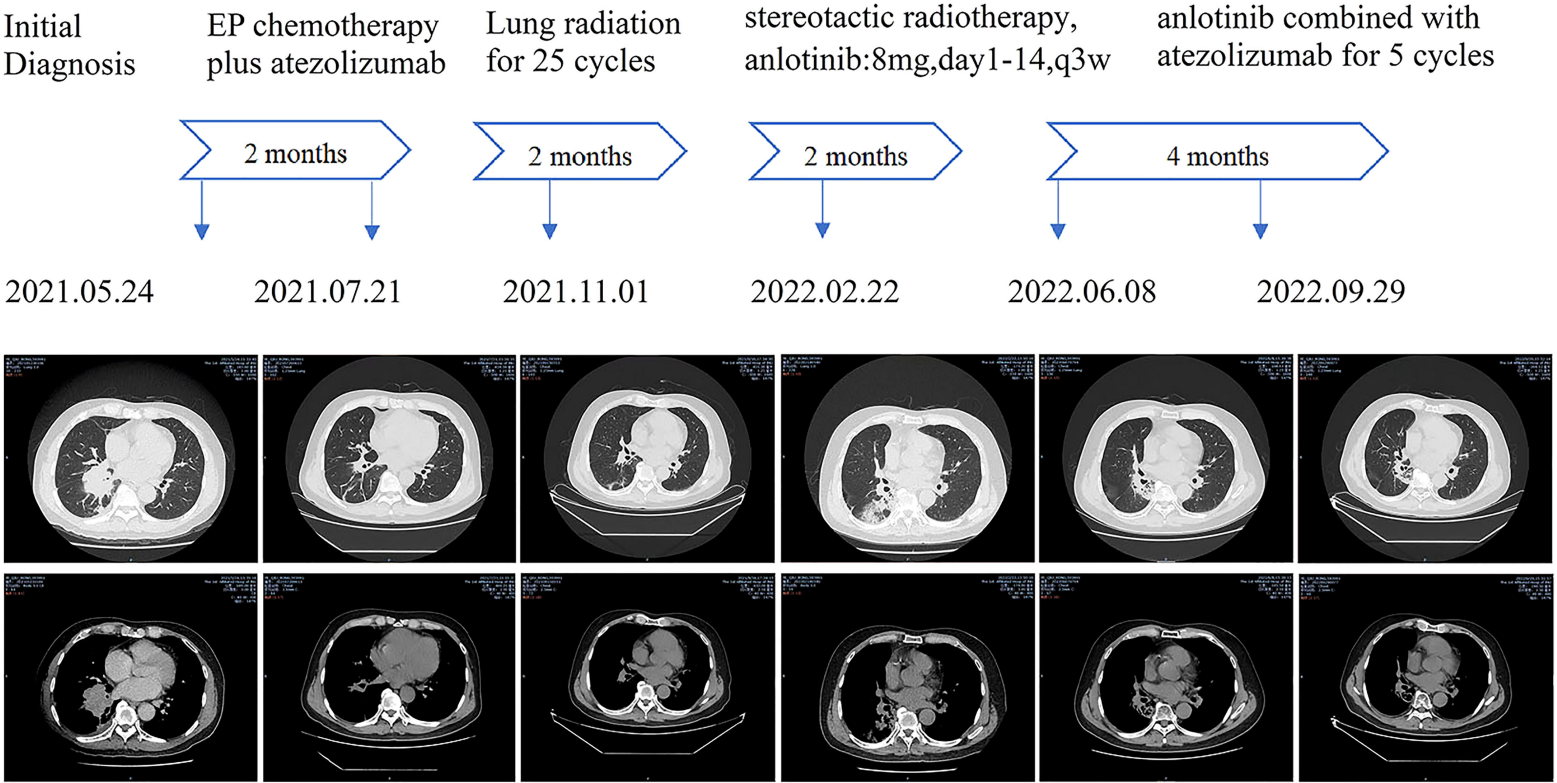
Chest CT images of primary lung noules at different time points.

In May of 2021, a 62-year-old male was admitted for having cough and expectoration for three months. The patient had been evaluated of a right lung lobe mass and obstructive pneumonia at another hospital. He has a forty-year history of smoking and chronic bronchitis. He denied any contagious disease history and was not vaccinated recently, but his wife and children were HBsAg positive. Chest computed tomography (CT) indicated a 5.0×4.5×4.0 cm uneven-density shadow in the right lower lobe, and the basal bronchi of the right upper and lower lobes were occluded (**Figure 1**). The nodular shadow that was evaluated pathologically by transbronchial biopsy and immunohistochemical analysis: Syn (+), CgA(-), CD56(+), and Ki-67(90%+). Enhanced CT scanning revealed many enlarged lymph nodes in the mediastinum and right hilus pulmonis. No metastasis was detected in the adrenal gland, bone scan, or cerebral magnetic resonance imaging (MRI).

The patient was diagnosed with LS-SCLC based on the aforesaid examination results and had received 150mg etoposide(day1-day3), 500mg carboplatin(day1), and 1200mg atezolizumab per day as first-line therapy since July 4th, 2021.

**Figure 2:**
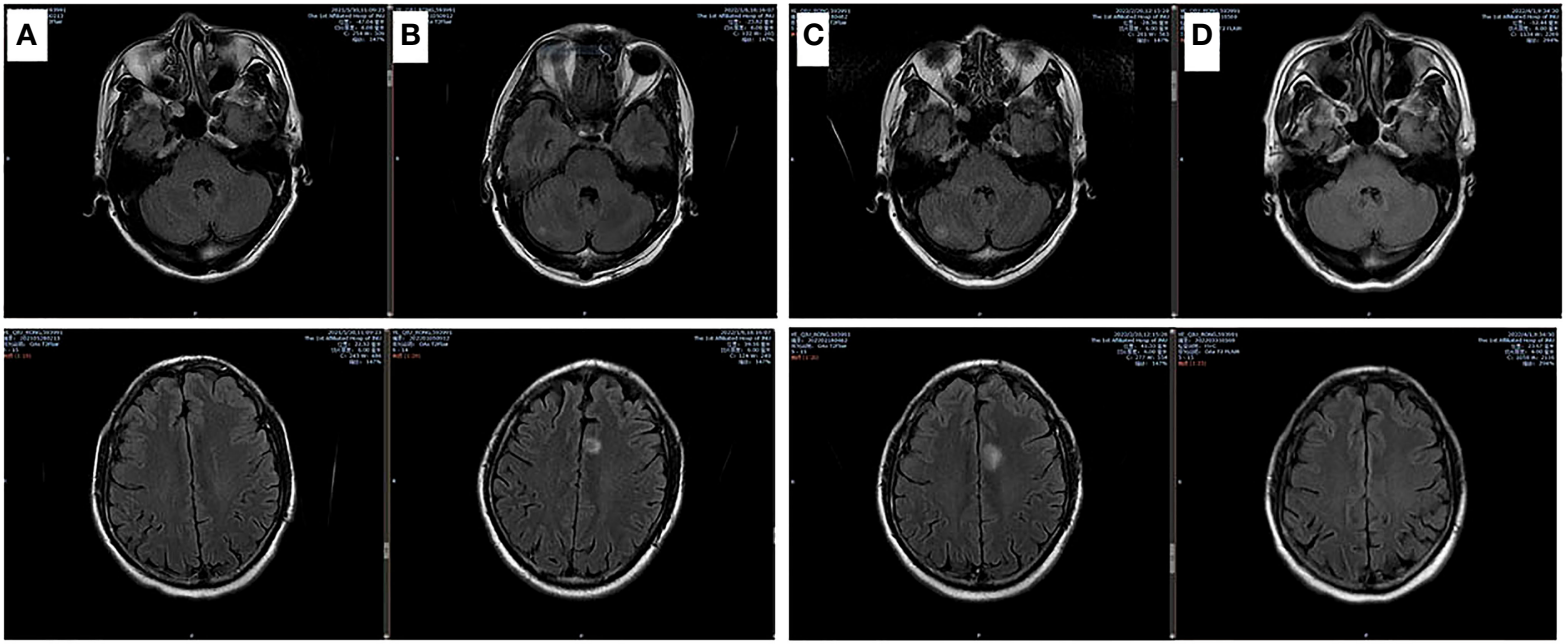
Brain MRI images of involved matastasis images. **(A)** Base line; **(B)** After chemotherapy for 5 cycles and lung radiation for 25 cycles; **(C)** 1 month after newly BMs; **(D)** 1 month after brain radiation and anlotinib treatment.

After 4 cycles of EC chemotherapy and atezolizumab immunotherapy, the size of the patient’s right lower lobe shadow was visibly reduced to roughly 2.0×1.3cm, the patient reached stable disease (SD). The test showed the HBsAb level was 447.26mIU/ml but the HBsAg was negative on August 18th, 2021.After the fifth dose of 1200 mg atezolizumab cure, he got chest radiation (60Gy/30f). It was halted due to cough and radiation-related pneumonitis. After approximately one week of Methylprednisolone treatment, the symptoms were released. On January 7th, 2022, the routine cranial MRI revealed metastases in the left frontal lobe and right cerebellar hemisphere, with nodes measuring 1.1×0.8cm and 0.5×0.5cm, respectively. In the meantime, larger right pulmonary nodule (sized 2.1×1.9cm) and patchy high-density plot region are dispersed throughout the right lung lobe (**Figure 1**). The situation of progressive disease (PD) was evaluated based on the examination. The patient was re-assigned to receive two gamma knife treatments. On April 1st, 2022, a cranial MRI revealed scant indications of persistent metastases in earlier lobes (**Figure 2**). On the following days, the patient was referred for 1200mg atezolizumab and monthly combination therapy of atezolizumab (1200mg d1/q4w) and anlotinib (8mg d1-d14/q3w). Surprisingly, the HBsAb level raised to over 10000mIU/ml but the HBsAg was still negative on April 28th, and the reexamination showed the HBsAb level still maintained over 10000mIU/ml without HBsAg detected on September 28th,2022. Timeline of the treatment was shown in **Table 1**.

**Table 1:** Timeline of the treatment.

Time	Medical examination	Evaluation of disease	Therapeutic schedule
2021.05.22	Chest contrast-enhanced CT, brain contrast-enhanced MRI, whole-body bone scan, bronchoscopy	Small-cell lung cancer of the right lung at ES stage	Lung lesions biopsy
2021.06.04-2021.08.19	Chest and brain CT	Partial response	Etoposide: 200 mg on day 1 to 3, carboplatin:500mg on day 1, atezolizumab 1200mg on day 1.
2021.09.18	Chest and brain CT	Stable disease	atezolizumab 1200mg
2021.11.01-2022.01.06	–	Stable disease	Lung radiation
2022.01.06	Brain MRI	Progressive disease	
2022.02.22-2022.02.25	Brain MRI	Progressive disease	stereotactic radiotherapy
2022.03.02	–	Stable disease	anlotinib:8mg,day1-14,q3w
2022.04.02	Brain MRI	Partial response	atezolizumab 1200mg
2022.05.09-2022.09.29	Chest CT	Partial response	atezolizumab 1200mg q4w, anlotinib:8mg,day1-14,q3w

## 3 Discussion

SCLC is a type of neuroendocrine cancer that is distinguished by its rapid progression, ease of recurrence, and high invasiveness (15). One of the major factors influencing the survival and life quality of SCLC patients is the rate of brain metastases, which can reach 60% to 80% in patients with a survival of more than 2 years (16). Recent research showed the addition of anti-angiogenic to anti-PD-1 therapy could improve the outcomes in ES-SCLC. But the basis support of anlotinib combined with PD-1/PD-L1 inhibitors in ES-SCLC has not been explored sufficiently.

The effect of combination therapy may be related to complex tumor angiogenesis and variable tumor immune microenvironment. Vascular endothelial growth factors (VEGFs) family participate in growth and permeation of blood vessels, which can promote tumor angiogenesis (17, 18). Anlotinib is the only antiangiogenic drug currently approved for SCLC patients in the third line treatment. Anlotinib inhibits numerous targets such as VEGFR2-3, fibroblast growth factor receptor1-4(FGFR1-4), and platelet derived growth factor receptor (PDGFR) (8). Anlotinib resulted in impaired tumor angiogenesis and normalization of remaining blood vessels. In addition, the use of anti-PD-1 antibody extended the period of vascular normalization. Further research revealed that anti-CD4+T cells reduced survival time (19). This finding suggests that PD-1 inhibitor may reverse the early exhausted CD4+ T cells. It has been reported that CD4+ T cells mediate vessel normalization and infiltration of immune cells in a IFNγ-dependent pattern (20). And IFNγ-mediated induction of PD-L1 was viewed as a prominent signaling (21). Anti-PD-L1 antibodies contributed to tumor apoptosis and improved sensitivity to chemotherapies (22). Besides, anlotinib can downregulate expression of PD-L1 on vascular endothelial cells to inhibit tumor growth (23). Therefore, the combination therapy enhanced the activation and infiltration of CD4+ T cells, moreover, provided normal vessel for transporting PD-1/PD-L1 antibodies.

Meanwhile, ICIs also can induce irAEs referring to multiple organs, limiting their application in various cancers (24). These irAEs are not organ specific and commonly involve in skin, liver, gastrointestinal tract, lungs, skeletal muscle, endocrine system (25). Among HBV-related hepatitis patients, HBV reaction is a serious complication. HBV clearance depend on specific T cell response like secreting CD8+ and CD4+ T cells (26). But the co-expression molecules on T cells including PD-L1 can impair the T cell response and result in T cells exhaustion. PD-L1 antibody may reverse exhausted T cells and reboot HBV specific T cells immune response (27). Based on that, there were clinical trials regarding on the potentials of PD-L1 antibody functionally cured HBV infection. At the 2023 annual meeting of the Asian Pacific Association for the Study of the Liver, the functional cure of chronic hepatitis B was firstly announced. After 24 weeks oral presentation of ASC22 (Envafolimab), a subcutaneous PD-L1 antibody, three subjects achieved functional cure until the end of follow-up.

In this case, we firstly reported a HBsAg-negative patient with sustained increase of HBsAb after medication of PD-L1. However, even after vaccination, most people’s protective HBsAb levels are insufficient. It could be linked to delayed immune response, latent infection, and immune tolerance. In addition to T cell immunity, innate immune cells involving monocytes and natural killer cells also play roles in immune response during HBV infection (28, 29). HBV-induced PD-L1 on monocytes interacts with PD-1 ligands on NK cells to educate the differentiation of IL-10+ regulatory NK cells. Moreover, NK-reg cells can inhibit the CD4+ and CD8+ T cells activation, resulting in exhausted immune response within the liver microenvironment (30). The patient we reported may have had low dose HBV infection while contacting his family. This could be one of the reasons he produced protective HBsAb. After anti-PD-L1 treatment, CD4+and CD8+T cells were reactivated, reversing exhausted immune cells, and causing constant increase of HBsAb. This phenomenon suggests a potential method for increasing HBsAb level and a possible solution for reducing chronic HBV infection. But the further mechanism is needed to be explored especially in HBV immune response.

## 4 Conclusion

Based on the above analysis of inner mechanism, the combination of anlotinib and atezolizumab for ES-SCLC is available. We described an ES-SCLC with BMs patients who obtained favourable prognosis. After receiving PD-L1 antibody treatment, the HBsAg-negative patient’s HBsAb level increased persistently. The unusual occurrence of this patient encouraged us to find out theoretical support. The effects on tumor vessel structure and cancer immune microenvironment should not be overlooked. PD-L1 antibodies have potential to functionally cure HBV infection and induce protective HBsAb. We hope that more research will be conducted for chronic HBV infection patients with cancers.

## Data availability statement

The raw data supporting the conclusions of this article will be made available by the authors, without undue reservation.

## Ethics statement

Written informed consent was obtained from the individual(s) for the publication of any potentially identifiable images or data included in this article.

## Author contributions

GC and TT contributed to the collection and assembly of clinical data. XC conducted the disease analysis and provided summary. All authors contributed to the article and approved the submitted version.

## References

[1] Welter S Aigner C Roesel C . The role of surgery in high grade neuroendocrine tumours of the lung. J Thorac Dis (2017) 9(Suppl 15):S1474–S83. doi: 10.21037/jtd.2017.01.60 PMC5690951 29201450

[2] Rudin CM Brambilla E Faivre-Finn C Sage J . Small-cell lung cancer. Nat Rev Dis Primers (2021) 7(1):3. doi: 10.1038/s41572-020-00235-0 33446664 PMC8177722

[3] Zugazagoitia J Paz-Ares L . Extensive-stage small-cell lung cancer: First-line and second-line treatment options. J Clin Oncol (2022) 40(6):671–80. doi: 10.1200/JCO.21.01881 34985925

[4] Song Y Fu Y Xie Q Zhu B Wang J Zhang B . Anti-angiogenic agents in combination with immune checkpoint inhibitors: A promising strategy for cancer treatment. Front Immunol (2020) 11:1956. doi: 10.3389/fimmu.2020.01956 32983126 PMC7477085

[5] Zhang X Zeng L Li Y Xu Q Yang H Lizaso A . Anlotinib combined with PD-1 blockade for the treatment of lung cancer: A real-world retrospective study in China. Cancer immunol immunother CII (2021) 70(9):2517–28. doi: 10.1007/s00262-021-02869-9 PMC10991983 33566148

[6] Yu L Xu J Qiao R Han B Zhong H Zhong R . Efficacy and safety of anlotinib combined with PD-1/PD-L1 inhibitors as second-line and subsequent therapy in advanced small-cell lung cancer. Cancer Med (2022) 12(5):5372–5383. doi: 10.1002/cam4.5360 PMC10028028 36250532

[7] Lopes-Coelho F Martins F Pereira SA Serpa J . Anti-angiogenic therapy: Current challenges and future perspectives. Int J Mol Sci (2021) 22(7):3765. doi: 10.3390/ijms22073765 PMC8038573 33916438

[8] Shen G Zheng F Ren D Du F Dong Q Wang Z . Anlotinib: a novel multi-targeting tyrosine kinase inhibitor in clinical development. J Hematol Oncol (2018) 11(1):120. doi: 10.1186/s13045-018-0664-7 30231931 PMC6146601

[9] Han B Li K Wang Q Zhang L Shi J Wang Z . Effect of anlotinib as a third-line or further treatment on overall survival of patients with advanced non-small cell lung cancer: The ALTER 0303 phase 3 randomized clinical trial. JAMA Oncol (2018) 4(11):1569–75. doi: 10.1001/jamaoncol.2018.3039 PMC6248083 30098152

[10] Deng P Hu C Chen C Cao L Gu Q An J . Anlotinib plus platinum-etoposide as a first-line treatment for extensive-stage small cell lung cancer: A single-arm trial. Cancer Med (2022) 11(19):3563–71. doi: 10.1002/cam4.4736 PMC9554443 35526266

[11] Chen Q Li Y Zhang W Wang C Yang S Guo Q . Safety and efficacy of ICI plus anlotinib vs. anlotinib alone as third-line treatment in extensive-stage small cell lung cancer: A retrospective study. J Cancer Res Clin Oncol (2022) 148(2):401–8 doi: 10.1007/s00432-021-03858-2. PMC8800903 34797416

[12] Hussaini S Chehade R Boldt RG Raphael J Blanchette P Maleki Vareki S . Association between immune-related side effects and efficacy and benefit of immune checkpoint inhibitors - a systematic review and meta-analysis. Cancer Treat Rev (2021) 92:102134. doi: 10.1016/j.ctrv.2020.102134 33302134

[13] Okiyama N Tanaka R . Immune-related adverse events in various organs caused by immune checkpoint inhibitors. Allergol Int (2022) 71(2):169–78. doi: 10.1016/j.alit.2022.01.001 35101349

[14] Zhang X Zhou Y Chen C Fang W Cai X Zhang X . Hepatitis b virus reactivation in cancer patients with positive hepatitis b surface antigen undergoing PD-1 inhibition. J Immunother Cancer (2019) 7(1):322. doi: 10.1186/s40425-019-0808-5 31753012 PMC6873745

[15] Yang S Zhang Z Wang Q . Emerging therapies for small cell lung cancer. J Hematol Oncol (2019) 12(1):47. doi: 10.1186/s13045-019-0736-3 31046803 PMC6498593

[16] Rittberg R Banerji S Kim JO Rathod S Dawe DE . Treatment and prevention of brain metastases in small cell lung cancer. Am J Clin Oncol (2021) 44(12):629–38. doi: 10.1097/COC.0000000000000867 34628433

[17] Viallard C Larrivée B . Tumor angiogenesis and vascular normalization: Alternative therapeutic targets. Angiogenesis (2017) 20(4):409–26. doi: 10.1007/s10456-017-9562-9 28660302

[18] Zhao Y Guo S Deng J Shen J Du F Wu X . VEGF/VEGFR-targeted therapy and immunotherapy in non-small cell lung cancer: Targeting the tumor microenvironment. Int J Biol Sci (2022) 18(9):3845–58. doi: 10.7150/ijbs.70958 PMC9254480 35813484

[19] Su Y Luo B Lu Y Wang D Yan J Zheng J . Anlotinib induces a T cell-inflamed tumor microenvironment by facilitating vessel normalization and enhances the efficacy of PD-1 checkpoint blockade in neuroblastoma. Clin Cancer Res (2022) 28(4):793–809. doi: 10.1158/1078-0432.CCR-21-2241 34844980 PMC9377760

[20] Tian L Goldstein A Wang H Ching Lo H Sun Kim I Welte T . Mutual regulation of tumour vessel normalization and immunostimulatory reprogramming. Nature (2017) 544(7649):250–4. doi: 10.1038/nature21724 PMC5788037 28371798

[21] Noguchi T Ward JP Gubin MM Arthur CD Lee SH Hundal J . Temporally distinct PD-L1 expression by tumor and host cells contributes to immune escape. Cancer Immunol Res (2017) 5(2):106–17. doi: 10.1158/2326-6066.CIR-16-0391 PMC5510474 28073774

[22] Kornepati AVR Vadlamudi RK Curiel TJ . Programmed death ligand 1 signals in cancer cells. Nat Rev Cancer (2022) 22(3):174–89. doi: 10.1038/s41568-021-00431-4 PMC9989967 35031777

[23] Liu S Qin T Liu Z Wang J Jia Y Feng Y . Anlotinib alters tumor immune microenvironment by downregulating PD-L1 expression on vascular endothelial cells. Cell Death disease (2020) 11(5):309. doi: 10.1038/s41419-020-2511-3 32366856 PMC7198575

[24] Williams KC Gault A Anderson AE Stewart CJ Lamb CA Speight RA . Immune-related adverse events in checkpoint blockade: Observations from human tissue and therapeutic considerations. Front Immunol (2023) 14:1122430. doi: 10.3389/fimmu.2023.1122430 36776862 PMC9909476

[25] Ramos-Casals M Brahmer JR Callahan MK Flores-Chávez A Keegan N Khamashta MA . Immune-related adverse events of checkpoint inhibitors. Nat Rev Dis Primers (2020) 6(1):38. doi: 10.1038/s41572-020-0160-6 32382051 PMC9728094

[26] Fanning GC Zoulim F Hou J Bertoletti A . Therapeutic strategies for hepatitis b virus infection: Towards a cure. Nat Rev Drug discovery (2019) 18(11):827–44. doi: 10.1038/s41573-019-0037-0 31455905

[27] Liu L Hou J Xu Y Qin L Liu W Zhang H . PD-L1 upregulation by IFN-α/γ-mediated Stat1 suppresses anti-HBV T cell response. PloS One (2020) 15(7):e0228302. doi: 10.1371/journal.pone.0228302 32628668 PMC7337294

[28] Iannacone M Guidotti LG . Immunobiology and pathogenesis of hepatitis b virus infection. Nat Rev Immunol (2022) 22(1):19–32. doi: 10.1038/s41577-021-00549-4 34002067

[29] Aliabadi E Urbanek-Quaing M Maasoumy B Bremer B Grasshoff M Li Y . Impact of HBsAg and HBcrAg levels on phenotype and function of HBV-specific T cells in patients with chronic hepatitis b virus infection. Gut (2022) 71(11):2300–12. doi: 10.1136/gutjnl-2021-324646 PMC9554084 34702717

[30] Li H Zhai N Wang Z Song H Yang Y Cui A . Regulatory NK cells mediated between immunosuppressive monocytes and dysfunctional T cells in chronic HBV infection. Gut (2018) 67(11):2035–44. doi: 10.1136/gutjnl-2017-314098 PMC6176520 28899983

